# Early metabolic response in sequential FDG-PET/CT under cetuximab is a predictive marker for clinical response in first-line metastatic colorectal cancer patients: results of the phase II REMOTUX trial

**DOI:** 10.1038/s41416-018-0152-4

**Published:** 2018-07-02

**Authors:** Anne Katrin Berger, Stephan Lücke, Ulrich Abel, Georg Martin Haag, Carsten Grüllich, Annika Stange, Mareike Dietrich, Leonidas Apostolidis, Angelika Freitag, Claudia Trierweiler, Carl von Gall, Jennifer Ose, Frederik Giesel, Tim Frederik Weber, Florian Lordick, Uwe Haberkorn, Dirk Jäger

**Affiliations:** 1 Department of Medical Oncology, National Center for Tumor Diseases (NCT), University Hospital Heidelberg, Heidelberg, Germany; 2 National Center for Tumor Diseases (NCT), Trial Center, German Cancer Research Center (DKFZ), Heidelberg, Germany; 3 Department of Surgery, University Hospital Dresden, Dresden, Germany; 4 Siemens Medical Solutions USA, Knoxville, USA; 5 Cancer Institute University of Utah, Salt Lake City, USA; 6 Department of Nuclear Medicine, University Hospital Heidelberg, Heidelberg, Germany; 7 Department of Diagnostic and Interventional Radiology, University Hospital Heidelberg, Heidelberg, Germany; 8 University Cancer Center Leipzig (UCCL), University Medicine Leipzig, University of Leipzig, Leipzig, Germany

**Keywords:** Colorectal cancer, Cancer imaging

## Abstract

**Background:**

To assess the predictive value of early metabolic response (ΔSUV) after short-term treatment with first-line cetuximab in patients (pts) with RAS-wt metastatic colorectal cancer (mCRC).

**Methods:**

In this prospective phase II study, RAS-wt mCRC pts received a single-agent cetuximab run-in therapy of 2 weeks. ΔSUV was assessed with FDG-PET/CT on days 0 and 14. Early clinical response (ECR) was evaluated with CT on day 56 after treatment with FOLFIRI-cetuximab. Primary endpoint was the predictive significance of ΔSUV for ECR. Secondary endpoints were PFS (progression free survival), OS and the influence of ΔSUV on survival.

**Results:**

Forty pts were enroled and 33 pts were evaluable for the primary endpoint. The CT response rate was 57.6%. For responders, ΔSUV was significantly higher (*p* = 0.0092). A significant association of ΔSUV with ECR was found (*p* = 0.02). Median PFS was 11.7 months and median OS was 33.5 months with a 1-year survival rate of 87.9%. ΔSUV was found to significantly impact the hazard for OS (*p* = 0.045).

**Conclusions:**

We demonstrate that cetuximab induces metabolic responses in mCRC pts. The study endpoint was met with the ΔSUV discriminating between responders and non-responders. However, these data should be validated in larger patient cohorts.

## Introduction

Currently, the median overall survival (OS) for patients with metastasised colorectal cancer (mCRC) is almost 2.5 years with response rates of 60% for the most active regimen.^1^ For the RAS-wildtype (wt) population, first-line anti-EGFR therapy is reported with improved response rates and increased resectability of metastases compared to anti-VEGF treatment, whereas the impact on survival is discussed controversially.^2–4^ However, as not all RAS-wt patients respond to anti-EGFR therapy, clinical exploration and validation of further biomarkers for this collective is warranted.^5^ Usually, treatment response evaluation in mCRC is based on morphological size criteria of tumour lesions as measured with computed tomography (CT) or magnetic resonance imaging (MRI), requiring a systemic treatment period of at least 6 to 8 weeks. Changes in the tumoural glucose metabolism are thought to precede morphological changes, and ^18^F-fluorodeoxy-glucose-positron-emission-tomography/CT (FDG-PET/CT) has proven relevance in early predicting treatment response associated with improved survival in several solid tumour entities.^6–8^

We conducted a prospective phase II trial with sequential FDG-PET/CT before and after a 2-week run-in phase with single-agent cetuximab in RAS-wt first-line mCRC patients. We aimed to evaluate the predictive significance of early changes in FDG uptake under single-agent cetuximab for the first morphological response as measured on day 56 under treatment with the FOLFIRI-cetuximab regimen.

## Methods and patients

### Trial design

This open-label, non-randomised phase II clinical trial was conducted at the National Center for Tumor Diseases (NCT), Heidelberg, Germany. The protocol was approved by the national regulatory authorities according to the requirements of §40ff. AMG (German Drug Law). All aspects of the study were done in accordance with the declaration of Helsinki and the guidelines for Good Clinical Practice of the International Conference on Harmonization. An independent data monitoring committee monitored recruitment and safety.

### Patients

Patients aged ≥18 years with histologically confirmed mCRC were eligible for inclusion if they had no history of therapy with an EGFR targeting agent or chemotherapy for advanced disease, and measurable tumour lesions with a diameter no smaller than 1.0 cm. An ECOG-performance status of 0 or 1 was mandatory as well as life expectancy >12 weeks and adequate haematological, renal and hepatic function. A KRAS-wt status (exon 2) of the tumours was requested. As in 2014, the approval for cetuximab was restricted to patients with both KRAS- (exons 2, 3, 4) and NRAS- (exons 2, 3, 4) wt status, the inclusion criteria of this trial were adapted accordingly. Full inclusion and exclusion criteria are given in Supplement 1.

### Study treatment

Cetuximab was administered intravenously (i.v.) 400 mg/m^2^ on day 1 and 250 mg/m^2^ on day 8 as single-agent therapy within this study. For patients with any grade 3 or 4 toxicity (NCI-CTCAE), cetuximab could be withheld. For patients with severe skin reactions (≥grade 3), cetuximab therapy could be withheld until the reaction had resolved to grade 2. The duration of the study treatment was 2 weeks. Between day 14 and day 56, the patients were treated according to the FOLFIRI-cetuximab regimen as an active and approved first-line treatment for this patient cohort. According to the trial center’s clinical standards, the FOLFIRI-cetuximab regimen was applied every 2 weeks as follows: cetuximab 250 mg/m^2^ (days 1, 8), irinotecan 180 mg/m^2^ (day 1), folinic acid 400 mg/m^2^ (day 1), 5-fluorouracil 400 mg/m^2^ bolus (day 1) and 2400 mg/m^2^ for 46 h (day 1). Depending on the response on day 56, treatment was continued according to the choice of the responsible physician. It was recommended that, in case of response, treatment was continued with cetuximab and FOLFIRI until disease progression or patients were unable to tolerate the therapy.

### Imaging methodology and response analysis

#### FDG-PET-CT imaging

At baseline (day 0) and at day 14, patients received an FDG-PET/CT for evaluation of early changes in tumour glucose uptake. Patients had to fast at least 6 h prior to the i.v. application of the radiopharmakon, and the blood glucose level should not exceed 150 mg/dl. All patients received 20–40 mg butylscopolaminiumbromide i.v. to reduce bowl movements. A CT scan for attenuation correction was performed 1 h post tracer injection. Immediately after CT scanning, a whole-body PET was acquired in 3D (matrix: 164 × 164). For each bed position (16.2 cm, overlapping scale: 4.2 cm) we used 4 min. acquisition time with a 15.5 cm field of view (FOV). The emission data were corrected for randoms, scatter and decay. Reconstruction was conducted with an ordered subset expectation maximisation algorithm (OSEM) with 4 iterations/8 subsets and Gauss-filtered to a transaxial resolution of 4.2 mm at full width at half maximum (FWHM). Attenuation correction was performed using the low dose non-enhanced CT data. PET and CT were performed using the same protocol for every patient on a BIOGRAPH-6 PET/CT scanner (Siemens, Germany). For calculation of the standardised uptake value (SUV), circular regions of interest were drawn around areas with focally increased uptake in transaxial slices and automatically adapted to a three-dimensional volume of interest at a 70% isocontour. At baseline, a diagnostic contrast-enhanced CT scan of the chest, abdomen and pelvis during the portal venous phase was included in the FDG-PET/CT protocol.

### CT scanning and radiological assessment of response

At day 56 (±4 days), evaluation of morphological response was performed with a routine contrast-enhanced portal venous phase CT scan covering chest, abdomen and pelvis (Philips Brilliance iCT, The Netherlands). Response assessment was performed using the Response Evaluation Criteria in Solid Tumors version 1.1 (RECIST 1.1^9^). Early clinical response was defined as partial remission (PR) or complete remission (CR) according to RECIST 1.1.

### Statistical design and sample size

The primary objective was the evaluation of the predictive significance of relative changes in SUV (ΔSUV) in FDG-PET/CT during short-term single-agent treatment with cetuximab. Specifically, ΔSUV was defined as 100×(SUV_baseline_–SUV_d14_)/SUV_baseline_. All study patients were analysed for whom ΔSUV and response measurement were available. Secondary objectives included the duration of PFS and OS as well as the influence of ΔSUV and early clinical response on PFS and OS. PFS was defined as the time from study entry to objective tumour progression or death from any cause, whichever occurred first. PFS data were censored on the date of the last tumour assessment on study for patients who did not have primary disease progression (PD) and who did not die while on study. Patients lacking any tumour assessment after the first treatment administration had their PFS time censored on the date of first treatment administration with a duration of 1 day. OS was defined as the time from study entry to death from any cause. For living patients, time to death was censored at the time of last contact. The trial was designed to detect an AUC of ΔSUV = 0.8 with a power of 84.1% requiring *n* = 35 evaluable patients. For detailed sample size calculation, see Supplement 2.

### Statistical methods

The null hypothesis that ΔSUV has no predictive power for early clinical response was tested using the Wilcoxon rank-sum test (2-sided, *α* = 5%) comparing responders (CR, PR) with non-responders. The degree of discrimination was quantified by means of the area under the ROC curve (AUC). The primary analysis was performed on all patients who were evaluable for the primary endpoint and who met the original inclusion criteria. The association of ΔSUV with the clinical response was analysed by logistic regression. Standard methods for survival analysis (Kaplan-Meier estimates of the survival curves,^10^ Cox proportional hazards regression^11^) were used in the analysis of time-to-event endpoints PFS and OS. The Wilcoxon rank-sum test was used to compare the ΔSUV values of clinical responders vs. clinical non-responders. The Wilcoxon signed-rank test was used to test changes in SUV from baseline to day 14 for statistical significance (2-sided, *α* = 5%).

## Results

### Recruitment and patient characteristics

**Fig. 1:**
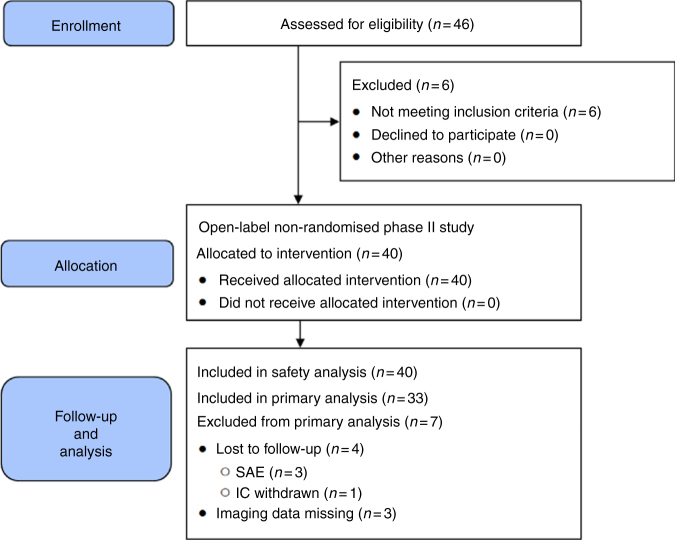
Patient disposition: CONSORT flow diagram

From February 2011 to November 2014, 40 patients were enroled at our centre. All patients received the study medication and were analysed for safety (*safety group*). Four patients discontinued the trial (three patients due to adverse events (AEs), one patient withdrew consent). Two patients missed CT scan on day 56, and one patient did not show metabolic activity in FDG-PET/CT. Thus, 33 patients (82.5%) were evaluable for the primary analysis (*per-protocol-group*). Information about the patient disposition is summarised in Fig. 1, which was prepared according to the current version of the CONSORT statement.

### Patient population

Median age of patients was 62 years (range 32–78), and 15 patients were ≥65 years. In the *per-protocol-group*, 16 patients (48.5%) had distant metastases restricted to the liver. Two and three patients had metastases restricted to lung or lymph nodes, respectively. Twelve patients (36.4%) had involvement of more than one organ site. Eleven patients (33.3%) later underwent a resection approach for metastastic disease. Twenty-two patients (66.7%) received second-line chemotherapy, and 11 patients (33.3%) underwent three or more treatment lines. The first 26 patients were enroled under the original inclusion criteria (KRAS-wt exon 2), and 14 patients complied with full NRAS- and KRAS-wt status. For patients included prior to restriction of RAS criteria, an additional retrospective analysis for NRAS and full KRAS status was done whenever possible. For three patients of the *per-protocol-group*, extended RAS data were missing (two patients without NRAS analysis and one patient with missing NRAS exon 4 analysis). In four patients, primarily unrecognised mutations in KRAS exons 3 or 4 or NRAS were found. Patients’ baseline characteristics are given in Table 1. For detailed information on disease extent and resectability potential, see Supplement 3.

**Table 1:** Patient characteristics

Baseline characteristic	*N* (%) (*n* = 40)
Age	
Years (median, range)	62 (32–78)
Sex	
Male	31 (77.5)
Female	9 (22.5)
Race	
Caucasian/White	38 (95.0)
Oriental/Asian	2 (5.0)
ECOG PS	
0	28 (70.0)
1	12 (30.0)
Primary Site	
Rectum	20 (50.0)
Colon	18 (45.0)
>1 Primary site	2 (5.0)
Site of metastases	
Liver	34 (85.0)
Lung	10 (25.0)
Other	13 (32.5)
Interval between first diagnosis and metastatic disease	
Days (median, range)	66.5 (0–2068)

### Safety

The rate of AEs under cetuximab treatment was well within expectations with the skin and the gastrointestinal tract being the most affected organ classes in 16 (40%) and 26 patients (65%), respectively. Fourteen patients experienced an AE of grade 3 or higher of which hypersensitivity reactions were the most frequent (three patients, 7.5%). One patient was reported with an atypical pneumonia resulting in death without suspected relation to study treatment. AEs were collected and graded using the National Cancer Institute Common Terminology Criteria for Adverse Events, v 3.0.

### Metabolic imaging and early morphological response

CT scans on day 56 showed a PR in 17 patients (51.5%) and a CR in two patients (6.1%). Twelve patients (36.4%; including all four patients with post-hoc identified RAS mutations) had a stable disease (SD) and two patients (6.1%) had a PD. Thus, the early morphological response rate as defined in this trial (PR+CR) in the *per-protocol-group* was 57.6% under treatment with FOLFIRI-cetuximab. With sequential FDG-PET/CT, the median SUVmax was 8.9 (range 3.0–19.8) at baseline and 6.0 (range 1.9–15.0) on day 14. The median relative reduction in SUV (ΔSUV) at day 14 for all patients was 30.9% (range −33.3 to 65.7). For early morphological responders, the median ΔSUV was 42.3% (range −14.3 to 65.7) vs. a median ΔSUV of 20.3% (range −33.3 to 50.4) in the group of non-responders. The four non-wt patients had a median ΔSUV of 10.7% (range −8.9 to 20.9%), see Supplementary Figure 1.

### Proof-of-princle and effect size: the predictive power of ΔSUV

**Fig. 2:**
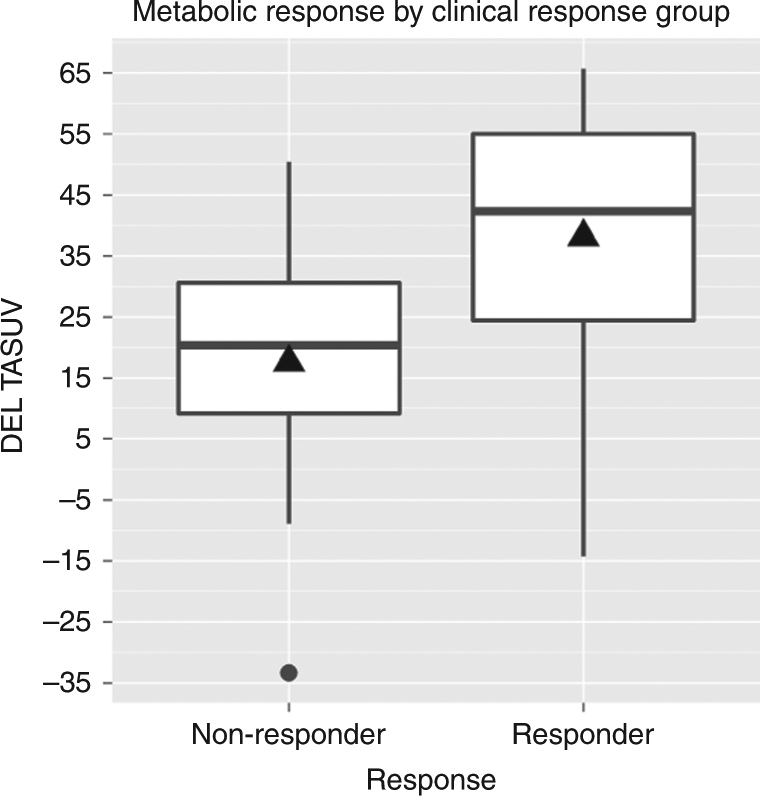
Distribution of ΔSUV by response with group means (diamond), *per-protocol-group*

**Fig. 3:**
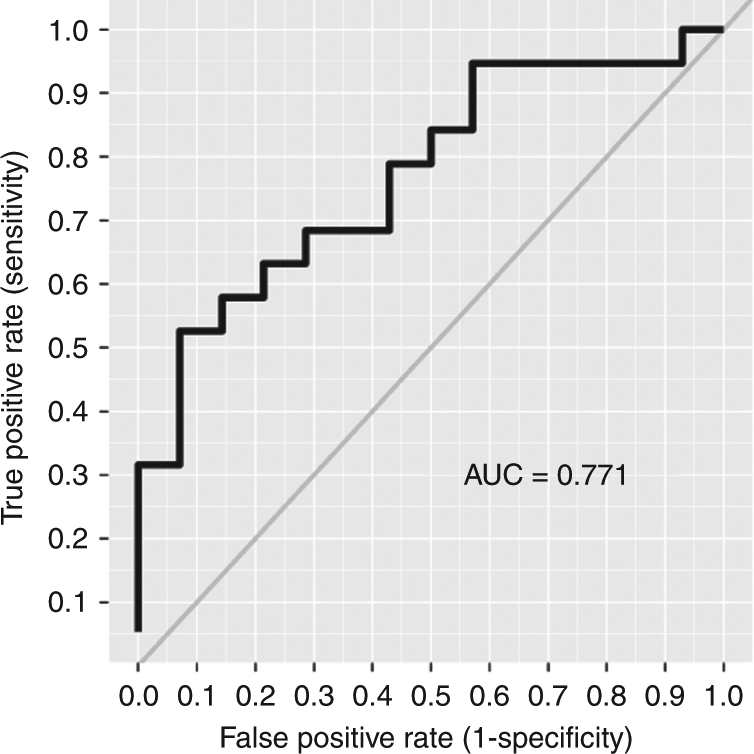
ROC analysis for ΔSUV with respect to early clinical response, *per-protocol-group*

**Fig. 4:**
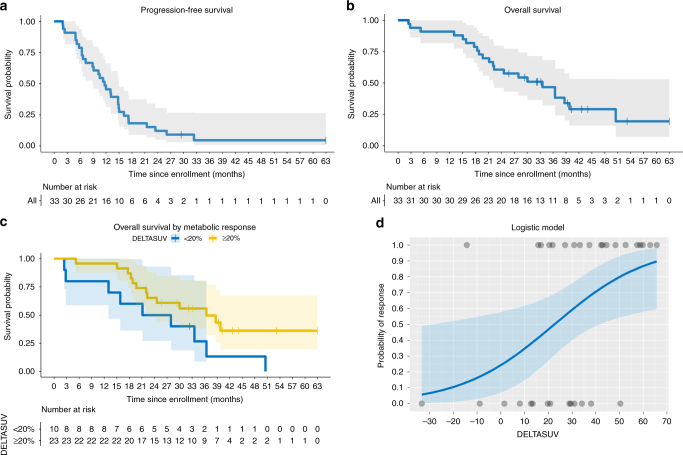
**a** Kaplan–Meier estimates of PFS with number of subjects at risk, *per-protocol-group*. **b** Kaplan–Meier estimates of OS with number of subjects at risk, *per-protocol-group*. **c** Kaplan–Meier estimates of OS by reduction in SUV (%), *per-protocol-group*. **d** Predicted probabilities of response for the logit model with 95% confidence bounds, *per-protocol-group*

The primary objective of this study was to evaluate the prognostic relevance of relative changes in SUV for early clinical response. The ΔSUV for responders was found to be significantly higher than for the group of non-responders, indicating that ΔSUV is—in principle—able to discriminate between non-responders and responders (*p* = 0.0092, Fig. 2). The degree of discrimination was quantified by means of the area under the ROC curve (AUC, Fig. 3). The AUC was determined to be 0.771 [95% CI: 0.609–0.933], indicating that the probability that a random responder has a higher value of ΔSUV than a random non-responder is 77.1%. Logistic regression analysis indicated a statistically significant association of ΔSUV with early clinical response (*p* = 0.02; OR = 1.052 [95% CI: 1.007–1.098]). The adapted logistic model can be used to predict the clinical outcome based on the measured relative change in SUV (Fig. 4d).

### Patient follow-up and survival times

Patients were followed up for at least 24 months. The median duration of follow-up for the *per-protocol-group* was 42.7 months with 31 patients (93.9%) experiencing disease progression. Two patients finished follow-up without event after 29.4 and 63.0 months, respectively. Median PFS was 11.7 months [95% CI: 7.3–14.8] (Fig. 4a) and the 1-year PFS rate was 45.5% [95% CI: 28.2–61.2]. Twenty patients (66.7%) died during follow-up. Median OS was 33.5 months [95% CI: 21.1–39.8] (Fig. 4b) with a 1-year survival rate of 87.9% [95%CI: 70.9–95.3]. ΔSUV was found to significantly impact the hazard for OS in univariable Cox regression analysis (*p* = 0.045; HR = 0.98 [95%CI: 0.95–0.999]). In an explorative analysis, a ΔSUV >20% (fitting with the EORTC recommendations for partial metabolic response^12^) was found to support the trend towards improved OS for metabolic responders (Fig. 4c).

## Discussion

Early treatment stratification for mCRC patients is relevant for both avoiding unnecessary toxicity and in terms of health care economics. Of note, modern antineoplastic treatments allow an increasing amount of *conversion therapies* in primarily irresectable mCRC cases, with an OS exceeding 5 years for the RAS-wt subgroup.^4^ A prolonged preoperative systemic treatment in these patients can raise the rate of surgical complications substantially, making a secondary curative approach impossible in the worst case scenario.^13–15^ Thus, oncologists are in need of reliable criteria for upfront identification of the most effective therapy for the patient and of tools that will allow for early response-adaptive treatment guidance. For radiologic assessment, early tumour shrinkage (ETS) was suggested as a potential predictive marker for long-term outcome in RAS-wt mCRC patients.^16^ Still, there are primary anti-EGFR non-responders in the RAS-wt collective, suggesting heterogeneity in EGFR signalling, and development of diagnostic tools predicting prognosis and therapy-response is of high interest. Innovative techniques such as *liquid biopsies* may provide a great potential for identification of further predictive molecular markers in this setting and may even allow for non-invasive monitoring of clonal dynamics. To date, prospective clinical validation is awaited, and recent guidelines do not recommend on molecular testing beyond extended RAS, BRAF and MSI-analysis.^17^ FDG-PET/CT is an imaging approach allowing metabolic response assessment with the SUV being the most widely applied semiquantitative parameter.^18^ For response assessment, changes in the SUV (ΔSUV) rather than the absolute SUV value have proven useful.^19, 20^ In mCRC, only few prospective studies have evaluated the use of FDG-PET/CT for response monitoring. In older series on mCRC patients, metabolic response correlated with objective response but not with survival.^8, 21, 22^ So far, to our knowledge, no data on the effect of EGFR-blockade are available.

Our results show as a proof-of-principle that short-term single-agent treatment with cetuximab induces a high rate of metabolic tumour response in mCRC patients. This finding is in good accordance with data for other solid tumours under inhibition of the EGFR pathway.^23, 24^ Concerning RAS analysis, our results are consistent with those from previous studies showing that patients with mutant tumours are unlikely to benefit from anti-EGFR therapy.^25^

The ΔSUV at day 14 in our cohort differed significantly between early morphological responders (defined as PR or CR) and non-responders. Thus, we could demonstrate that cetuximab induces (i) early relevant metabolic changes in mCRC that are (ii) predictive for later morphological response achieved by the combination of FOLFIRI plus cetuximab. Despite our rather small sample size, we found that ΔSUV had a significant impact on OS, suggesting that early metabolic response may hold prognostic value beyond prediction of early clinical response. The early FDG-PET/CT may prove useful as a tool for identification of mCRC patients benefitting from anti-EGFR treatment. It might serve as a complementary imaging strategy for rapid clinical verification of therapy-response, as needed for example in the *neoadjuvant* or *conversion* setting, even when an enlarged armamentarium of molecular signatures will be available. In non-responders, an early change or escalation of the regimen (FOLFOX + Bevacizumab or FOLFOXIRI + Bevacizumab) might be beneficial in this setting. However, the question if a FDG-PET/CT-guided algorithm will translate into improved survival times or improved resection rates can only be answered by larger and randomised trials. The additional costs of FDG-PET/CT should be noted, but would probably be well outweighed by saving a very costly, toxic and quality-of-life-reducing therapy. However, it must be considered that FDG-PET/CT is not yet available in all regions and to all patients.

In addition, our results show that early FDG-PET/CT assessment is feasible for single-agent-targeted therapies in only a short-time treatment phase. Thus, it may prove useful considering early drug development with the provided information on immediate effects on tumour cells facilitating the identification of substances qualifying for further clinical evaluation.

However, our data of this small prospective patient collective can only be viewed as a pilot. The prospective definition of clinical meaningful cut-off points for ΔSUV allowing prediction of response and survival was beyond the scope of the REMOTUX trial, and the ΔSUV cut-off of 20% comes from an exploratory analysis. Further validation in independent patient cohorts is required to confirm our results and to answer the remaining questions.

## Conclusion

Single-agent cetuximab treatment of only 14 days is able to induce metabolic responses in mCRC patients as measured by FDG-PET/CT. This early metabolic response is predictive for later morphologic response and may even predict survival times. These results could impact on future treatment stratification in RAS-wt mCRC patients with implementation of early FDG-PET/CT as an additional and helpful tool for optimal treatment guidance, especially in the *conversion* setting. However, futher prospective trials are needed to definitely answer the question of an FDG-PET/CT-guided treatment algorithm in mCRC and to date, its use cannot be recommended in a routine setting.

### Registration and protocol participate

The study was registered under EUDRA-CT: 2009-013279-23 and ISRCTN 75334801, and the protocol was published previously.^26^

## Electronic supplementary material

Supplementary figure 1

Inclusion criteria

Sample size calculation

Disease extent

## Availability of data and materials

All data are available via the corresponding author and the NCT trial centre.
