# Supplementary figures and images for: Early metabolic response in sequential FDG-PET/CT under cetuximab is a predictive marker for clinical response in first-line metastatic colorectal cancer patients: results of the phase II REMOTUX trial
Source: Br J Cancer. 2018 Jul 2;119(2):170–5. doi: 10.1038/s41416-018-0152-4 (PMC6048023; doi:10.1038/s41416-018-0152-4)

## Slide 1
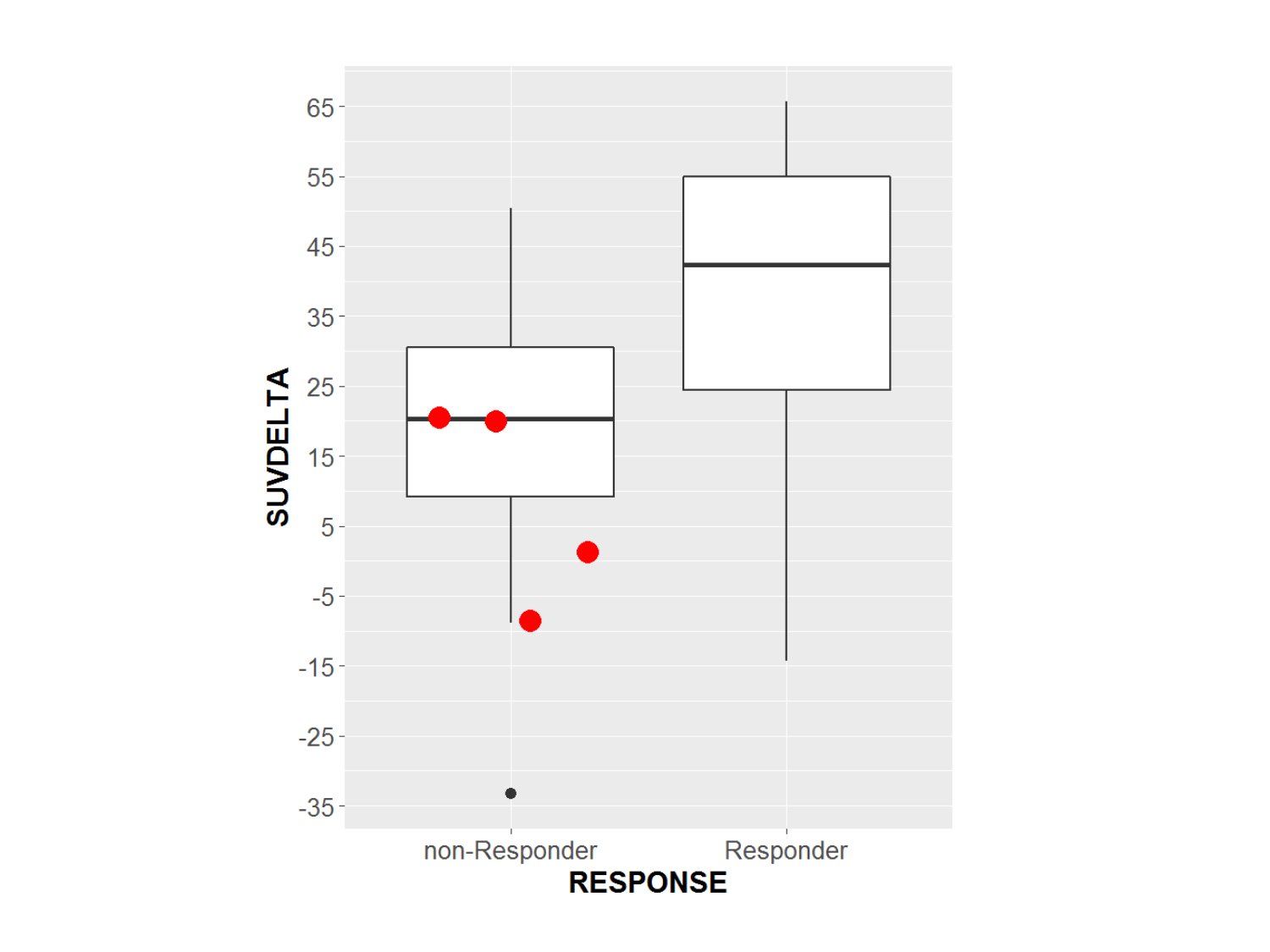

Supplement: Supplementary file 1 — Supplementary figure 1 [file 41416_2018_152_MOESM1_ESM.pptx]
