# Supplementary material for: Early metabolic response in sequential FDG-PET/CT under cetuximab is a predictive marker for clinical response in first-line metastatic colorectal cancer patients: results of the phase II REMOTUX trial
Source: Br J Cancer. 2018 Jul 2;119(2):170–5. doi: 10.1038/s41416-018-0152-4 (PMC6048023; doi:10.1038/s41416-018-0152-4)
Supplement: Supplementary file 2 — Inclusion criteria [file 41416_2018_152_MOESM2_ESM.docx]

**Supplements**

***Supplement 1:***

***Inclusion and exclusion criteria***

**Inclusion Criteria**

Patients meeting all of the following criteria were considered for enrollment:

- Histologically confirmed metastatic colorectal cancer
- RAS-wildtype status of the tumor (per Amendment No. 14)
- No history of therapy with an EGFR targeting agent
- No history of previous chemotherapy for advanced disease
- Measurable tumor lesion with a diameter no smaller than 1.0 cm detected by CT, MRI
  or ultrasound
- ECOG-performance status 0 or 1 or Karnofsky performance scale min. 70%
- Life expectancy > 12 weeks
- Age ≥ 18 years
- Adequate hematologic, renal and hepatic function
- Ability of the patient to understand the character and individual consequences of this
  clinical trial
- Written informed consent (must be available before enrollment in the trial)
- For women and men with childbearing potential adequate double barrier contraception,for women: negative pregnancy test
- Patients who are willing and able to comply with scheduled visits, treatment plan,
  laboratory tests, and other study procedures.

**Exclusion Criteria**

Patients presenting with any of the following criteria were not eligible:

- Any contraindications for chemotherapy according to the Folfiri regimen
- Non-curatively treated malignancy within the last 5 years
- Uncontrolled or insulin-dependent diabetes mellitus
- Evidence of CNS metastases
- Uncontrolled infection
- Significant cardiac disease (unstable angina pectoris or cardiac symptoms according to NYHA classification III or IV)
- Active serious illness that renders the patient unsuitable for study entrance or multiple
  blood sampling
- Pregnancy and lactation
- History of hypersensitivity to cetuximab or to any drug with similar chemical structure or to any excipient present in the pharmaceutical form of the investigational medicinal product
- Participation in other competing clinical trials or observation period of competing trials,respectively
- No patient was allowed to enroll in this trial more than once.
