# Supplementary material for: Early metabolic response in sequential FDG-PET/CT under cetuximab is a predictive marker for clinical response in first-line metastatic colorectal cancer patients: results of the phase II REMOTUX trial
Source: Br J Cancer. 2018 Jul 2;119(2):170–5. doi: 10.1038/s41416-018-0152-4 (PMC6048023; doi:10.1038/s41416-018-0152-4)
Supplement: Supplementary file 3 — Sample size calculation [file 41416_2018_152_MOESM3_ESM.docx]

***Supplement 2:***

***Sample size calculation***

The sample size/power calculations were based on the Wilcoxon rank sum test (two-sided testing; α=5%) in the form proposed by Noether [Noether G.E. (1987): Sample size determination for some common nonparametric statistics. JASA 82, 645-647] and implemented in the software package nquery advisor 6.01 [Statistical Solutions, Inc.: nquery advisor, v.01. Saugus, MA (2005)]. This form of the power calculation assumes that the alternative hypothesis is expressed as a probability P(Y>X), which, in our context, is the probability that a random responder has a higher value of ΔSUV than a random nonresponder. Note that the probability P(Y>X) is identical to the AUC of the ROC curve of ΔSUV with respect to the binary response. It should be observed that the group sizes of this test (i.e. the numbers of responders and nonresponders) cannot be determined in advance. Rather, the number of responders follows a binomial distribution with n = number of evaluable patients, and binomial probability r = probability of response. Therefore, the power of the test was determined as a weighted sum of the values of the power calculated for each possible constellation of group sizes of responders and nonresponders, the weights being the binomial probabilities that a particular constellation arises. Assuming that the values of ΔSUV in the groups of clinical responders and nonresponders, respectively, are represented by independent normally distributed variables with equal variances, and assuming that the true early clinical response rate of r is in the range 40% to 60%, a projected sample size of n=35 evaluable patients is sufficient to detect an AUC of ΔSUV (calculated with respect to response) of 0.8 with power 84.1% (the maximum power of 85.7% being attained for r=50%). It should be noted, however, that the true clinical response rate r is unknown. For r = 65% (instead of r = 60%) the estimated power for an AUC = 0.8 drops to 81.9%. Therefore, the slight overpowering if r is in the assumed range of values (40% to 60%) appears justified. Assuming that about 10% of the enrolled patients are not evaluable for the primary analysis, it is expected that the total number of patients to be enrolled in the study is 39. It is noteworthy that, under the assumptions specified above, 35 evaluable patients are also sufficient for detecting with power > 80% an AUC of 0.8 in the exploratory analysis of the main end point by means of a univariate logistic regression analysis as described in 10.5.4 (calculations based on 10000 computer simulations runs).
