# Supplementary material for: Early metabolic response in sequential FDG-PET/CT under cetuximab is a predictive marker for clinical response in first-line metastatic colorectal cancer patients: results of the phase II REMOTUX trial
Source: Br J Cancer. 2018 Jul 2;119(2):170–5. doi: 10.1038/s41416-018-0152-4 (PMC6048023; doi:10.1038/s41416-018-0152-4)
Supplement: Supplementary file 4 — Disease extent [file 41416_2018_152_MOESM4_ESM.docx]

**Supplement 3:**

Disease extent and metastatic sites

| **N (%); n=33** | **Metastatic site** | | | |
| --- | --- | --- | --- | --- |
|  | **Liver-only** | **Lung-only** | **Lymphatic-only** | **> 1 organ site** |
| **Total**  Diffus/multiple  ≤ 3 metastases | 16  13  3 | 2  -  - | 3  -  - | 12  -  - |
| **Resectability potential (initially)**  Upfront  None  Unlikely  In case of response | 1  3  6  6 | 1  1  -  - | -  1  1  1 | -  9  2  1 |
| **Resection** | 8 | 1 | 1 | 1 |
| **Ablative local treatment** | 1 |  | 2 | 1 |
